# 凝胶渗透色谱-光散射联用表征聚合物摩尔质量的实验教学改革

**DOI:** 10.3724/SP.J.1123.2023.12005

**Published:** 2024-08-08

**Authors:** Congde QIAO, Wenke YANG, Zhongwei LI, Qinze LIU, Jinshui YAO

**Affiliations:** 齐鲁工业大学(山东省科学院)材料科学与工程学院, 山东 济南 250353; School of Materials Science and Engineering, Qilu University of Technology (Shandong Academy of Sciences), Jinan 250353, China

**Keywords:** 凝胶渗透色谱-光散射联用, 验证性实验, 设计性实验, 教学改革, gel permeation chromatography coupled with light scattering (GPC-LS), confirmatory experiment, designing experiment, teaching reform

## Abstract

凝胶渗透色谱-光散射(gel permeation chromatography-light scatting, GPC-LS)联用是目前最常用的表征聚合物摩尔质量的方法之一,具有灵敏度高、结果准确等特点,在科学研究与生产实践中得到了广泛应用。“凝胶渗透色谱-光散射联用表征聚合物摩尔质量”是《高分子物理实验》课程中一个重要的教学内容。然而,目前的GPC-LS实验教学内容简单,缺乏深度。本文在该实验项目原有内容的基础上进行扩充,重新设计出多套实验项目:(1)选取商品化的聚苯乙烯为实验样品,利用GPC-LS对其摩尔质量、摩尔质量分布以及回转半径等分子结构参数进行表征;(2)选取两种分子结构参数接近的聚丙烯腈样品,借助质量微分分布曲线揭示这两种样品在摩尔质量分布中的细微差别;(3)选取一系列不同摩尔质量的聚乙二醇为实验样品,通过比较其色谱图分析摩尔质量的高低对色谱峰的影响;(4)选取3种不同的聚合物(聚丙烯腈、聚甲基丙烯酸甲酯、聚*β*-环糊精)样品,借助构象图对其分子链的构象进行分析。此外,本文对实验教学方法进行了改革,将被动学习转变为主动学习,提高了学生的自主学习能力。通过本实验项目的教学改革探索,使学生能更加全面地理解凝胶渗透色谱-光散射联用的原理及应用,开拓了学生的知识视野,激发了学生的学习热情,提升了实验教学效果。

## Abstract (en)

Gel permeation chromatography coupled with light scattering (GPC-LS) is among the most common methods for determining the molar masses of polymers. GPC-LS is widely used in polymer science research and has been adopted for many industrial applications owing to its high sensitivity, accuracy, and precision. The determination of polymer molar masses using GPC-LS is an important experimental component of the “Polymer Physics Experiments” course. However, the present GPC-LS experimental teaching content tends to be overly simplistic and lacking in depth. Herein, the original experimental content is expanded and multiple sets of experiments are redesigned: (1) Using commercial polystyrene as an experimental sample, the molar mass, molar mass distribution, radius of gyration, and other molecular structure parameters are determined using GPC-LS; (2) Using two polyacrylonitriles with similar molecular structure parameters, subtle differences in the molar mass distributions of the samples are explored using differential mass distribution curves; (3) By comparing the chromatograms of a series of polyethylene glycols with different molar masses, the effect of molar mass on chromatographic peaks is investigated; and (4) For three different polymers (polyacrylonitrile, poly(methyl methacrylate), and poly(*β*-cyclodextrin)), the polymer chain conformations are analyzed using conformation plots (i.e., radius of gyration vs. molar mass). In addition, the experimental teaching method is modified to convert passive learning into active learning, thereby improving the students’ self-directed learning ability. This experimental teaching reform will help students obtain a more comprehensive understanding of GPC-LS principles and applications, stimulate their enthusiasm for learning, and improve the teaching quality of the experimental course.

凝胶渗透色谱(gel permeation chromatography, GPC)是一种利用由惰性多孔凝胶装填而成的色谱柱将溶液中的高分子按照其流体力学体积大小进行分离的液相色谱技术^[1]^。分子尺寸大即摩尔质量高的组分先洗脱出来,分子尺寸小即摩尔质量低的组分后洗脱出来。其分离机理是基于熵驱动的高分子在多孔凝胶内外分配达到平衡的体积排除机理,因此人们将这类方法统称为体积排除色谱(size exclusion chromatography, SEC)。传统的GPC只能提供样品的摩尔质量信息,且受标样的影响较大^[2]^。而激光光散射能够准确表征聚合物样品的绝对摩尔质量,是研究高分子聚合反应的有力手段^[3]^。凝胶渗透色谱-光散射(GPC-LS)联用是表征聚合物摩尔质量与摩尔质量分布的最常用手段之一,具有快速、准确、灵敏度高、重复性好等特点,能够提供聚合物的数均摩尔质量、重均摩尔质量及摩尔质量分布等信息^[4⇓⇓-7]^。此外,光散射还可以提供高分子在溶液中的构象以及聚合物与溶剂分子间的相互作用信息。因此GPC-LS联用是研究聚合物分子结构的有效工具,已成为在分子水平研究聚合物结构与性能关系的常用手段^[8]^。

然而,在目前的《高分子物理实验》教学项目中,GPC-LS联用通常仅被用于表征聚合物样品的摩尔质量与摩尔质量分布,实验教学内容单一,缺乏创新性。本文围绕该技术在高分子结构表征中的应用,推出了多套可供学生选做的设计性实验项目,弥补了这方面的缺陷。通过对该实验教学项目中原有内容的扩展,使学生在掌握GPC-LS联用实验技术基础上,能够利用该技术深入地研究高分子的远程结构,即高分子链的构象及聚合物的摩尔质量,在分子水平上加强了对高分子结构与性能之间复杂关系的理解。此外,通过对实验教学方法的改革,开拓了学生的知识视野,提高了实验教学效果,加深了学生对GPC-LS联用技术原理的理解和应用,培养了学生的团队合作意识与创新能力。

## 1 GPC-LS联用实验教学内容设计

本文对该实验项目原有的教学内容进行了优化与扩充,围绕该技术在高分子结构表征中的应用,借助仪器操作软件ASTRA强大的数据分析功能,推出了多套设计性实验项目。其主要内容如下:1)选取商品化的聚苯乙烯为实验样品,利用GPC-LS对其摩尔质量、摩尔质量分布以及回转半径等分子结构参数进行表征;2)选取两种分子结构参数接近的聚丙烯腈(polyacrylonitrile, PAN)样品,借助质量微分分布曲线揭示这两种样品在摩尔质量分布中的细微差别;3)选取一系列不同摩尔质量的聚乙二醇(PEG)为实验样品,通过比较其色谱图分析摩尔质量的高低对色谱峰的影响;4)选取3种不同的聚合物(PAN、聚甲基丙烯酸甲酯(polymethyl methacrylate, PMMA)、聚*β*-环糊精(poly(*β*-cyclodextrin),poly(*β*-CD)))样品,借助构象图对其分子链的构象进行分析。

## 2 实验案例分析

围绕上述实验内容,本文设计了4个实验教学案例并进行了分析,如下:(1)聚苯乙烯分子结构参数的表征;(2)PAN摩尔质量分布的表征;(3)不同摩尔质量PEG的表征;(4)聚合物分子链构象的表征。通过对这些实验案例的分析,有助于深入理解摩尔质量、分子链尺寸及分子链构象等这些远程结构的物理意义及其相互关系。

### 2.1 仪器与试剂

凝胶渗透色谱-激光光散射联用(Wyatt,美国);包括Optilab T-rEX示差检测器与DAWN HELEOS-Ⅱ激光检测器。

聚苯乙烯、PEG与硝酸钠均购自国药集团化学试剂有限公司(上海);PAN、PMMA、聚*β*-CD与高纯水均由实验室自制;二甲基甲酰胺(DMF)、溴化锂与叠氮钠均由Sigma公司(美国)提供。

### 2.2 色谱条件

实验案例(1)(2)(4) 色谱柱:PLgel 5 μm MIXED-C+PLgel 5 μm MIXED-D柱(300 mm×7.5 mm, Agilent公司);柱温:60 ℃;样品溶液质量浓度:3~5 mg/mL;流动相:含0.05 mol/L LiBr的DMF;流动相流速:1.0 mL/min;进样量:100 μL。

实验案例(3) 色谱柱:OHpak SB-803HQ和OHpak SB-804HQ (300 mm×8.0 mm, Shodex);柱温:35 ℃;样品溶液质量浓度:3~5 mg/mL;流动相:含0.1 mol/L NaNO_3_和0.2 g/L NaN_3_的水溶液;流动相流速:0.5 mL/min;进样量:100 μL。

### 2.3 结果与讨论

#### 2.3.1 聚苯乙烯分子结构参数的表征

图1a给出了某聚苯乙烯样品的GPC-LS色谱图,可以看出,光散射检测与示差检测均在洗脱时间10~17 min出现一个峰。由于样品的多分散性及管路延迟等原因,这两个峰通常不会重合。此外,示差检测在洗脱时间18~20 min出现的小峰与倒峰均代表溶剂峰,在洗脱时间20 min后,样品全部从色谱柱中洗脱出来。找到样品峰后,需要借助一个模型拟合计算摩尔质量。对于常见的聚合物而言,最常用的模型为Zimm模型^[9]^,见式(1)。

**图1:**
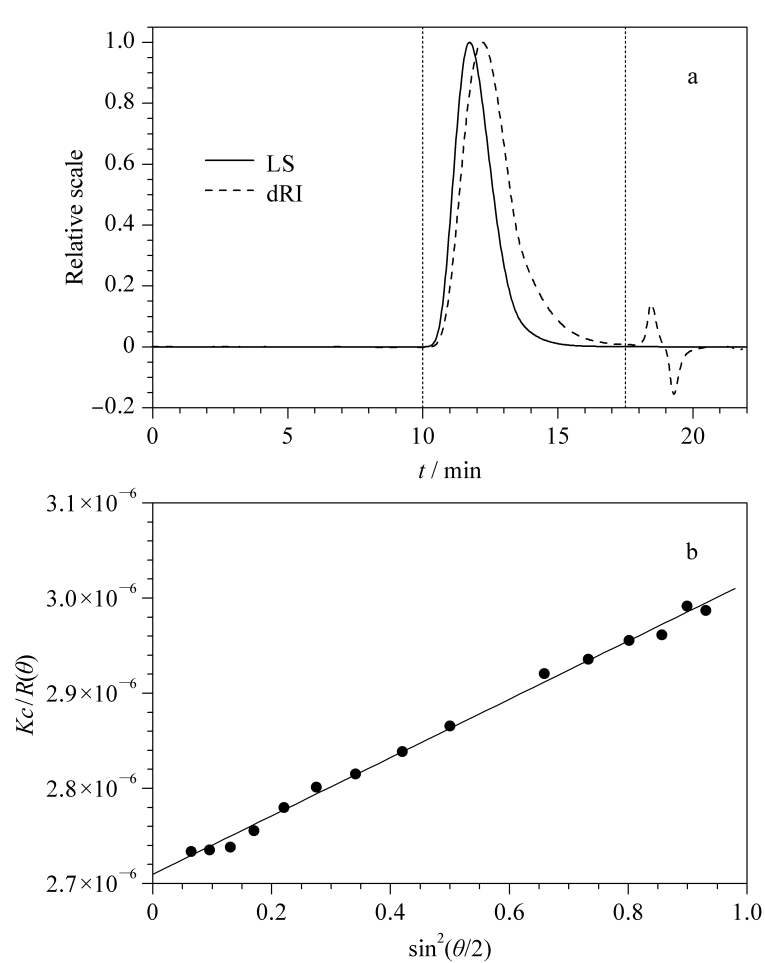
(a)聚苯乙烯样品的色谱图以及(b)光散射峰中某一级分的Zimm模式图


(1) $$\frac{Kc}{R(\theta )}=\frac{1}{M}\left(1+\frac{16\pi^{2}n^{2}}{3\lambda^{2}}R_{g}^{2}\sin^{2}\frac{\theta }{2}\right)$$

其中光学常数*K*=4*π*^2^*n*^2^/*N*_A_*λ*^4^(d*n*/d*c*)^2^, *n*和*λ*分别为溶剂的折光指数和入射光在真空中的波长,*c*为高分子的浓度,*R*(*θ*)是散射角为*θ*时测得的瑞利因子,*R*_g_为高分子的回转半径,*M*为摩尔质量。

由于散射峰的任何一点,其浓度都非常低(*c*约为10^-4^ g/mL),因此以*Kc/R*(*θ*)对sin^2^ $\frac{\theta }{2}$ 作图,其截距的倒数即为摩尔质量*M*,由斜率可得到分子的回转半径*R*_g_。图1b给出了样品峰中一个点(级分)的拟合计算结果,峰级分:354,拟合模型:Zimm,拟合度:1,洗脱时间:11.725 min,级分质量浓度:0.1565 mg/mL,级分的摩尔质量:(4.691±0.007)×10^5^ g/mol,级分的回转半径:(22.5±0.2) nm,样品的折光指数增量(d*n*/d*c*): 0.159 mL/g,拟合相关性:0.9957。

因此借助Zimm模型拟合可以得到样品光散射信号峰中每个级分的摩尔质量和回转半径。在此基础上就可以得到完整的样品摩尔质量-洗脱时间及回转半径-洗脱时间曲线(图2)。在图2数据的基础上,可以得到该聚苯乙烯样品的平均摩尔质量、摩尔质量分布系数以及回转半径等结构参数(表1)。

**图2:**
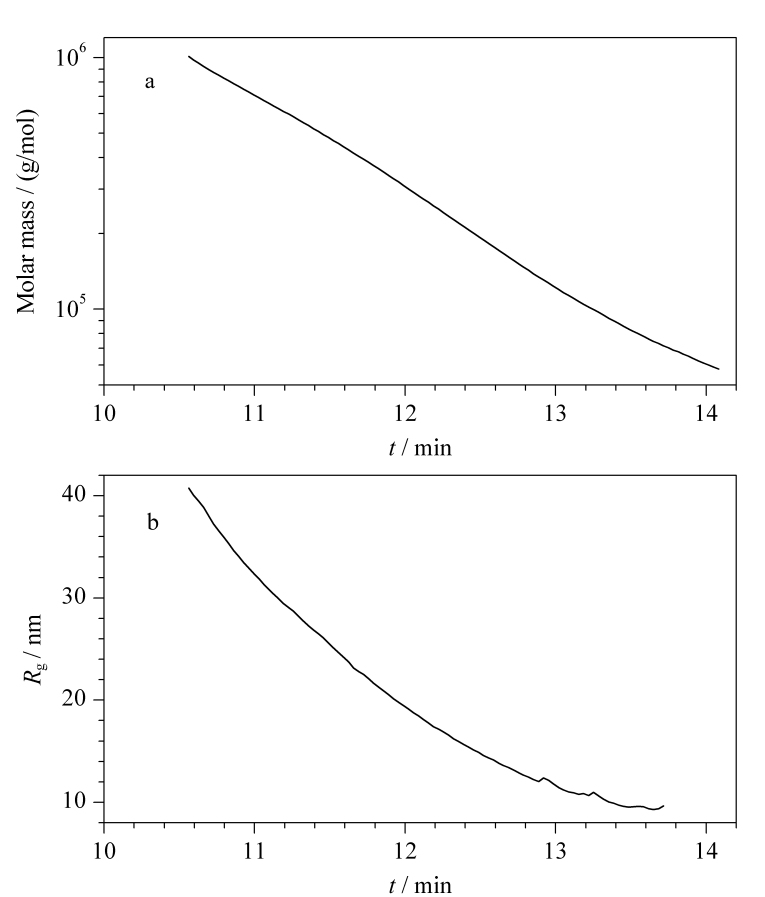
聚苯乙烯样品的(a)摩尔质量-洗脱时间及(b)回转半径-洗脱时间曲线

**表1:** 聚苯乙烯样品的GPC-LS测试项目及结果

Molecular structural parameter	Content	Result	Uncertainty
Molar mass moments/(g/mol)	M_n_	1.686×10^5^	0.2%
	M_p_	3.045×10^5^	0.1%
	M_w_	2.959×10^5^	0.1%
	M_z_	4.381×10^5^	0.3%
Polydispersity	M_w_/M_n_	1.755	0.2%
	M_z_/M_n_	2.599	0.4%
Radius of gyration/nm	R_gn_	13.7	4%
	R_gw_	17.9	2%
	R_gz_	22.2	1%

通过这个实验,学生可熟悉聚合物摩尔质量、摩尔质量分布以及回转半径等分子结构参数的测试方法及其物理意义,理解这些结构参数之间的关系及影响规律,加深对聚合物分子远程结构的理解,并初步掌握聚合物分子结构参数的表征及数据处理方法。

#### 2.3.2 PAN摩尔质量分布的表征

摩尔质量分布是聚合物分子结构的一大特色,聚合物本质上是由摩尔质量大小不同的同系物组成的混合物。该混合物的性能主要与某些摩尔质量级分的含量有关。因此,摩尔质量分布是聚合物最基本的结构参数之一,对其性能具有重要的影响^[10]^。聚合物摩尔质量分布的宽窄可以通过摩尔质量分布系数(*M*_w_/*M*_n_)进行表征。然而,单纯的摩尔质量分布系数只能给出摩尔质量分布的范围,并不能给出不同摩尔质量组分的含量。例如图3a中两种PAN样品的色谱图非常相似,其重均摩尔质量分别为90000 g/mol(PAN-1)与93000 g/mol(PAN-2),另外两种样品的摩尔质量分布也比较接近(PAN-1: 2.1; PAN-2: 2.2)。从平均摩尔质量与摩尔质量分布系数看,这两种PAN样品差别不大,然而二者的纺丝性能却显著不同。此时,需要借助质量微分分布曲线才能揭示这两种样品在摩尔质量分布中的细微差别。如图3b所示,PAN-1中摩尔质量为70000 g/mol左右的组分占的比例较高,而PAN-2中含量较高的组分的摩尔质量在90000 g/mol左右。由此可以推断,PAN-2的纺丝性能要优于PAN-1。因此借助质量微分分布曲线可以直观地显示出样品的摩尔质量分布差异。

**图3:**
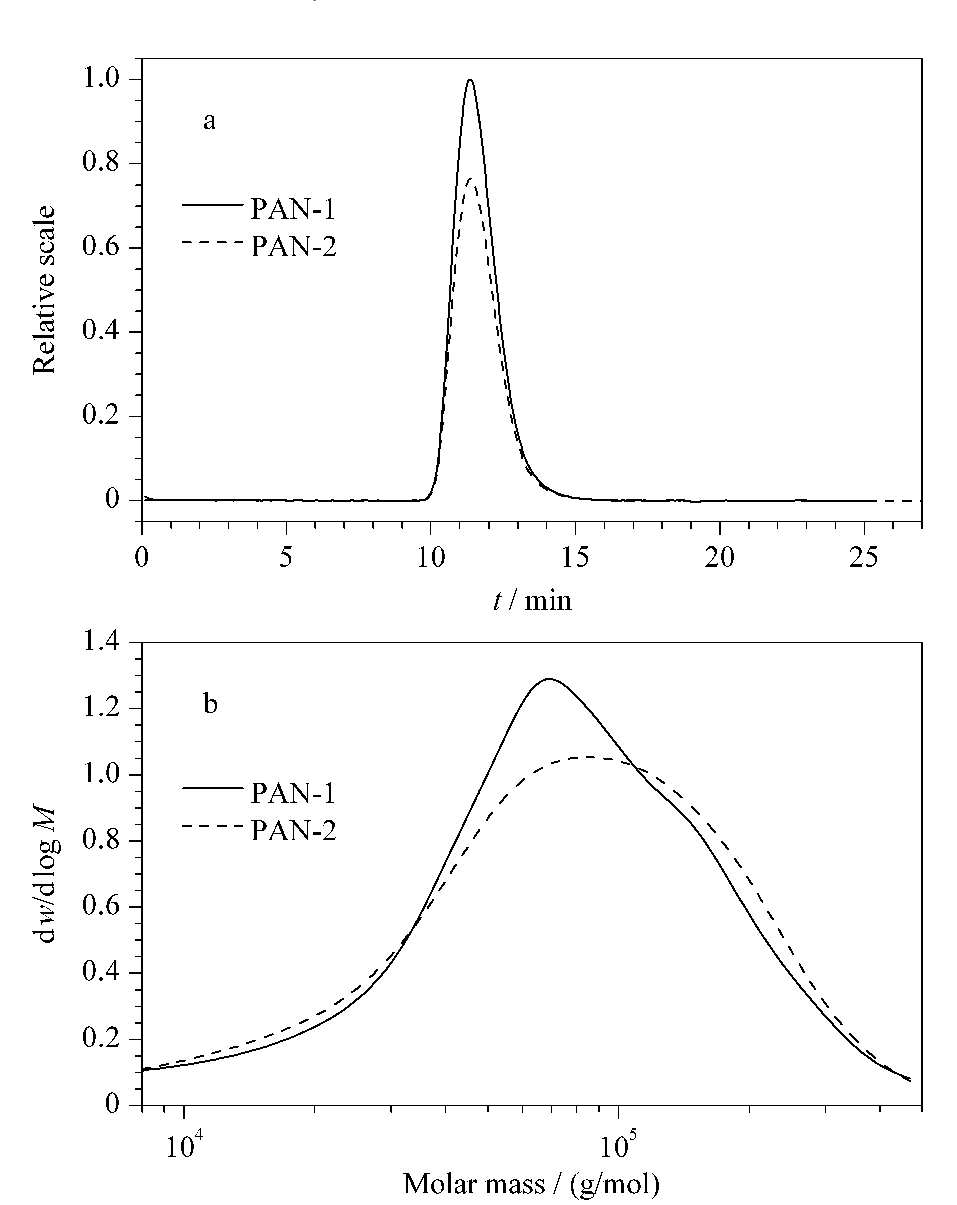
两种聚丙腈样品的(a)色谱图及其(b)质量微分分布曲线

通过该实验,学生可借助质量微分分布曲线更好地理解聚合物的摩尔质量分布,包括摩尔质量分布的宽度、分布的对称性以及摩尔质量集中的范围等。聚合物的摩尔质量分布与材料的力学性能如拉伸强度、冲击强度以及加工性能都有密切的关系。因此控制与改进聚合物的摩尔质量分布是高分子材料改性的重要途径。

#### 2.3.3 不同摩尔质量PEG的表征

学生在学习GPC机理的时候,很容易将组分的洗脱顺序与其摩尔质量的关系弄反。基于这种情况,为了能更直观地看出样品摩尔质量与其洗脱时间的关系,本文设计了该实验。如图4所示,不同摩尔质量的PEG样品的色谱峰位置显著不同。摩尔质量为20000 g/mol的PEG洗脱时间是30 min左右,随着样品摩尔质量的降低,其洗脱时间逐渐增加,当样品的摩尔质量降低至1000 g/mol时,其洗脱时间增加到38 min左右。此外,本实验选用的PEG是一种常用的水溶性高分子^[11]^,其生产厂家标注的摩尔质量与实验测定的摩尔质量存在一定的差异。不同摩尔质量PEG的测试结果见表2,从中可以看出,样品的重均摩尔质量与厂家标注的摩尔质量基本一致。

**图4:**
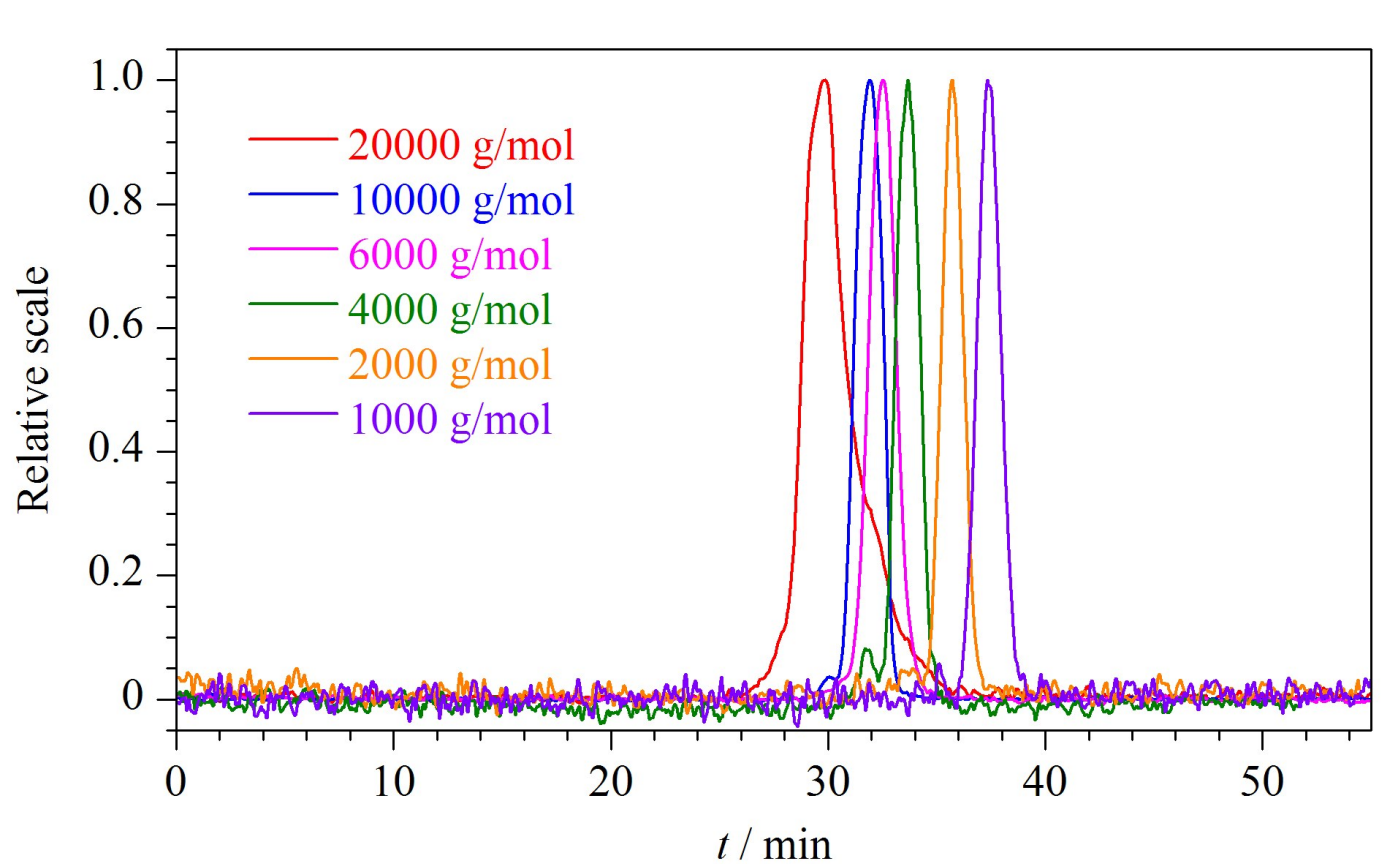
不同摩尔质量PEG的色谱图

**表2:** 不同摩尔质量PEG的测试结果

Label M_w_/(g/mol)	Determined M_w_/(g/mol)	Uncertainty
1000	1260	13%
2000	2210	12%
4000	4450	12%
6000	7760	9%
10000	8130	10%
20000	17430	6%

通过对不同摩尔质量的PEG色谱图的分析,学生可更直观地了解聚合物摩尔质量与其洗脱时间之间的关系,巩固对凝胶渗透色谱分离原理的认识,加深对高分子稀溶液理论的理解。

#### 2.3.4 聚合物分子链构象的表征

对于摩尔质量分布较宽的样品,通过测量经GPC分离后所得各级分的*M*与*R*_g_,借助ASTRA软件中的构象图(RMS conformation plot)建立*R*_g_与*M*之间的标度关系*R*_g_~*M^ν^*,从标度关系指数*ν*即可获得聚合物分子链构象的信息。研究表明,标度关系指数*ν*=1时大分子呈现棒状构象,*ν*=0.5~0.6对应着无规线团构象,*ν*=1/3则表明大分子紧密堆砌采取球形构象^[12]^。图5为PMMA、PAN和聚*β*-环糊精的构象图,拟合得到的标度关系指数分别为*ν*=0.56±0.03(PMMA)、0.58±0.02(PAN)和0.63±0.05 (poly(*β*-CD))。这一结果表明:PMMA、PAN和聚*β*-CD这3种不同化学结构的高分子在含0.05 mol/L LiBr的DMF中均呈现无规线团构象。与同为乙烯基聚合物的PMMA相比,相同摩尔质量下PAN的回转半径更大,这可由两者结构重复单元摩尔质量的差异来解释。相较于PMMA与PAN,相同摩尔质量下聚*β*-CD的回转半径最小,这是因为聚*β*-CD的结构重复单元*β*-环糊精具有紧凑的环状结构,整个聚合物链段所占的空间更小。

**图5:**
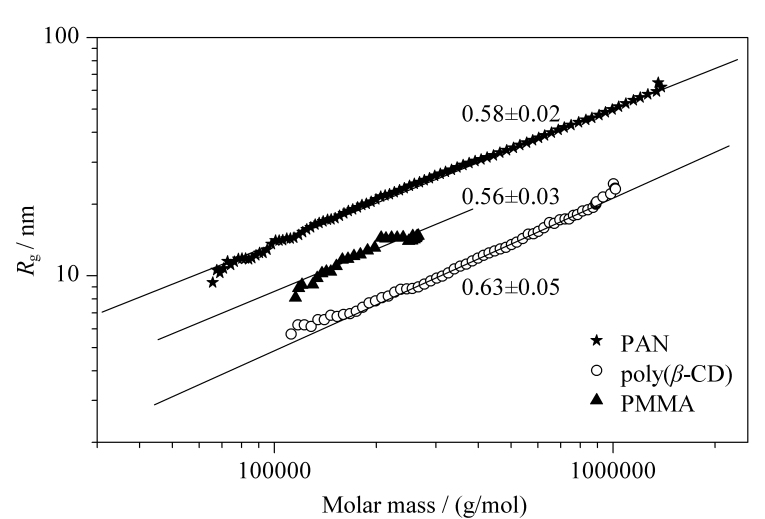
不同聚合物样品的回转半径与摩尔质量间的标度关系

通过对聚合物构象图的分析,学生可加深对聚合物分子链回转半径的概念及其测试方法的了解,熟悉高分子线团尺寸的变化规律及影响因素,促进对聚合物分子链构象的理解,初步掌握聚合物分子链构象的表征方法。

## 3 GPC-LS实验教学方法改革

在目前的GPC-LS联用实验教学中,由于教学方法单一,学生对GPC与LS的原理及其应用认识不足。学生通常只是在课堂上听老师讲述仪器的工作原理与操作规程,虽然对实验教学内容有一些了解,但通常都是处于一知半解的状态;实验结束后并不能及时复习巩固,而是在仓促提交实验报告后迅速转入其他课程的学习,直到下一次开始新的实验课程,从而导致教学效果不佳。针对这种大型测试设备而言,留给学生动手操作的时间很短,整个实验过程仿佛“过眼云烟”,难以保证教学效果。针对上述问题,本文创新教学模式,将被动学习转变为主动学习,提高了学生的自主学习能力。

实验开始前,老师通过布置预习报告作业提出若干问题,引导学生查阅相关资料,了解GPC-LS联用测聚合物摩尔质量的原理,影响GPC-LS测试的因素,色谱柱、溶剂,样品浓度的选择以及温度对摩尔质量的影响等。让同学们带着问题来做实验,做到“有备而来”,激发了学生的学习兴趣,提高了学习的主观能动性。

由于受到实验设备与实验学时的限制,每人只能完成一部分实验内容。基于这种现实情况,为提高课堂教学效率,我们将学生分成若干小组,每个小组完成不同条件下的实验项目。在实验教学中,对学生实验过程进行全程跟踪,引导学生进行正确的实验操作以及对实验现象的观察与分析。实验结束后对所有实验数据进行共享,实验教学内容得到了丰富。

实验结束后以小组为单位提交实验报告,并对整个实验项目包括实验目的、实验原理、实验设备、实验过程、数据处理、实验结果分析等进行论述,并推选代表展示PPT以汇报答辩的形式交流讨论各种实验因素对GPC-LS联用测试的影响。

## 4 教学效果评价

这种创新研究型实验的设计极大丰富了实验教学内容。在实验实施过程中,将多个知识点进行有机整合,使学生对GPC-LS联用的测试原理理解得更加形象生动。通过本实验项目的教学改革探索,开拓了学生的知识视野,激发了学生对GPC-LS联用技术的学习兴趣,加深了学生对于高分子溶液基本理论以及摩尔质量、摩尔质量分布、回转半径与构象等基本概念的理解。此外,丰富了高分子物理知识体系,提高了学生实验技能以及综合运用高分子物理基本理论分析和解决复杂高分子工程问题的能力,为学生的进一步学习奠定了基础。
